# Commentary: Dapagliflozin Mediates Plin5/PPARα Signaling Axis to Attenuate Cardiac Hypertrophy

**DOI:** 10.3389/fphar.2022.854593

**Published:** 2022-04-25

**Authors:** Zengying Liu, Ningxin Zhang, Bin Zhou, Yan Xu

**Affiliations:** Department of Nephrology, The Affiliated Hospital of Qingdao University, Qingdao, China

**Keywords:** microarray, bioinformatics analysis, differentially expressed genes, fold change, false discovery rate

Recently, we read the article published in frontiers in Pharmacology on 23 September 2021 written by Yu et al. (2021) with great interest: “Dapagliflozin Mediates Plin5/PPARα Signaling Axis to Attenuate Cardiac Hypertrophy.” In this article, the authors explored the molecular mechanism of dapagliflozin (DAPA) to relieve cardiac hypertrophy by conducting gene microarray analysis to identify differentially expressed genes (DEGs) and subsequently performing signaling pathway enrichment analysis. The study is of vital significance. However, we believe bioinformatics analysis has some weak points in the original article.

In the original article, microarray analysis was used to detect differentially expressed genes (DEGs) among the abdominal aortic constriction (AAC) group, AAC + DAPA group, and the sham group. The authors applied “fold change >1.5 or fold change <−1.5” as the threshold for significant differential expression. In the “Data Availability Statement” section of the original article, we noticed that the microarray GSE183120 that the authors analyzed was publicly available at the GEO database (https://www.ncbi.nlm.nih.gov/geo/query/acc.cgi?acc=GSE183120). On the GEO website, we could see only one sample in each of the groups depicted as sham, AAC, and AAC + DAPA, which means the *p*-value could not be generated with only one sample per group. Thus, the identification of DEGs may not be statistically significant. What is more, it is noteworthy that the platform shown in the GEO database of GSE183120 is “GPL23038 [Clariom_S_Mouse] Affymetrix Clariom S Assay, Mouse (Includes Pico Assay)” rather than what the authors mentioned in the section of “Microarray Profiling,” “Affymetrix GeneChip Primeview™.” Herein, we re-analyzed the microarray GSE183120 using “Transcriptome Analysis Console” (TAC software, version 4.0, Thermo Fisher Scientific) and produced different results. TAC4.0 is a software developed for researchers with no or little experience in bioinformatics, and it is suitable for Affymetrix microarray transcriptome analysis. Consistent with the original article, we also applied “fold change >1.5 or fold change <−1.5” as the threshold for significant differential expression, and other settings were default. We found that 2021 genes changed significantly, with 945 genes upregulated and 1,076 genes downregulated in the AAC + DAPA group compared with the AAC group, which was similar to the results of the original article. On the contrary, we were failed to reproduce fold change values of more than half of the representative genes, as shown in Table 1. In the results of our analysis (Table 2), representative genes showed the opposite direction in sham/AAC (sham vs. AAC comparison). For example, *STAT1* was upregulated in the AAC group compared with the sham group in our analysis but downregulated in the original article. Furthermore, a totally different fold change value of *STAT1* in AAC + DAPA/AAC (AAC + DAPA vs. AAC comparison) was obtained through our analysis. However, it experienced the same change direction as the original article.

**TABLE 1:** Fold changes of representative genes shown in the original article.

Gene name	Fold changes	
	Sham/AAC	AAC + DAPA/AAC
*STAT1*	3.227	−7.945
*Plin5*	−1.636	1.558
*Pdk4*	−75.584	18.896
*HMGCS2*	−134.364	9.646
*Cxcr4*	2.014	−2.099
*Tap1*	2.056	−1.853
*IGF1*	1.879	−2.071
*Sly*	6.916	−8.754
*Uty*	188.706	−206.5
*Rgs7*	−4.199	1.753
*Thy1*	−1.705	1.705
*Sdc2*	−1.879	1.879
*Vcp*	−2.189	1.919
*Gpx3*	−2.173	2.099
*Shb*	−2.25	1.84

**TABLE 2:** Fold changes of representative genes analyzed using TAC4.0 software.

Gene name	Fold changes	
	Sham/AAC	AAC + DAPA/AAC
*STAT1*	−3.22	−2.02
*Plin5*	1.64	1.56
*Pdk4*	75.54	18.88
*HMGCS2*	134.5	9.66
*Cxcr4*	−2.01	−2.1
*Tap1*	−2.05	−1.85
*IGF1*	−1.88	−2.08
*Sly*	−6.9	−8.76
*Uty*	−188.73	−206.53
*Rgs7*	4.19	1.75
*Thy1*	1.71	1.71
*Sdc2*	1.88	1.88
*Vcp*	2.18	1.92
*Gpx3*	2.19	2.1
*Shb*	2.25	1.83

All in all, it seems that the authors failed to conduct a hypothesis test and correction for DEGs. What is more, the screening process of DEGs needs thousands of hypothesis tests, resulting in an accumulation of false positives. Thus, the amplified false positives from such multiple hypothesis tests need to be corrected. Hence, it is essential to calculate the adjusted *p*-value in differential expressed genes analysis to control FDR using more suitable statistical methods such as Benjamini–Hochberg or some other algorithms.

Specialized high-level microarray analysis methods such as *Limma* (Linear Models for Microarray Analysis) (Ritchie et al., 2015; Law et al., 2016) for statistical analysis of DEGs is what we would like to recommend; setting the cutoff of absolute log_2_ FC > 1.5 and FDR < 0.05 (Zhang et al., 2019) is a more reliable approach to identify DEGs and enriched pathway appropriately.

In conclusion, it is the basis for further analysis, such as signaling pathway enrichment, to use optimal statistical approaches (Chrominski and Tkacz, 2015) and obtain accurate and convincing results of DEGs. We are looking forward to receiving further explanations about the data analysis from the authors and believe these considerations and innovations in data analytical and statistical approaches would benefit the data-intensive transcriptomics research (Liu et al., 2021).
